# Cultural Expressions of Koro Syndrome: A Case Series From North India

**DOI:** 10.7759/cureus.96981

**Published:** 2025-11-16

**Authors:** Shubham Sharma, Arpit Jaiswal

**Affiliations:** 1 Psychiatry, Rohilkhand Medical University, Bareilly, IND

**Keywords:** anxiety disorder, case series, culture bound syndrome, india, koro syndrome

## Abstract

Koro syndrome is a culture-bound psychiatric condition that is characterized by panic that one's genitals are pulling inward and that they are likely to die or develop sexual dysfunction. Southeast Asia has historically documented the epidemic and acute forms of Koro syndrome, while day-to-day practitioners continue to observe sporadic forms.

We report three clinical cases of young adult men (22-27 years old) from North India who presented with distressing, overvalued ideas of penile retraction associated with moderate to severe anxiety (Hamilton Anxiety Rating Scale scores: 20-27). Each presented with a characteristic cultural or psychosocial precipitant: exposure to cold in a factory laborer, masturbatory guilt and work-related stress in a software engineer, and a traditional remedy (use of cow dung) in a construction laborer. All the patients exhibited impaired socio-occupational performance, with normal physical examinations and investigations. Treatment consisted of selective serotonin reuptake inhibitors, short-term anxiolytics, psychoeducation, and supportive psychotherapy.

On short-term follow-up (from two weeks to one month), the three patients exhibited partial to significant improvement of anxiety and functional recovery with improved insight into the psychological etiology of the symptom.

This case series illustrates that Koro syndrome continues to be a clinical entity within contemporary Indian psychiatry, occurring beyond epidemic contexts and influenced by diverse cultural triggers. There should be recognition of such presentations and the use of culturally appropriate methods that include a combination of drug therapy, psychoeducation, and psychotherapy.

## Introduction

Koro syndrome is a psychiatric disorder that is characterized by an extreme fear that one's genitals will retract into the body, ultimately leading to death [1]. It may also be termed as culture-bound syndrome or culture-specific syndrome that combines both psychiatric and bodily symptoms for a given societal or cultural context. Koro syndrome has been especially reported within the Southeast Asian region. The name "Koro" has been derived from the Malay word "kura," meaning "tortoise," and metaphorically compares the retraction of the genitals (specifically the penis) with a tortoise's withdrawal of its head inside the shell. Koro has also been called Jhinjhinia Bemar within India, especially within Assam and sections of West Bengal [2].

Traditionally, Koro has been classified as Primary Koro, which occurs spontaneously without any associated psychiatric or physical illness, and associated psychiatric or physical illness, and Secondary Koro, which is seen in association with other psychiatric conditions such as anxiety or depressive disorders. Although it predominantly affects men, similar experiences have been described among women, who may report fears of breast retraction or genital inversion [3].

It was listed in the Diagnostic and Statistical Manual of Mental Disorders, fourth edition, under the category of culture-bound syndromes [4]. As with the worldwide anxiety among young males regarding penis size and being representative of masculinity and potency, such penile shrinkage may induce a great deal of anxiety. As we find in various cultures, the belief that penile shrinkage would result not only in erectile dysfunction and/or sterility but also in death should the genitals retract completely and "disappear. This accounts for emergency management exercised by such people during panic-like attacks of Koro, where holding on to the penis manually or by specific instruments provides slight relief.

In classical or cultural Koro syndrome, three symptoms need to be present, which are perception of a sharp retraction of the penis in males, sudden panic-like attack, and acute fear of impending danger, most commonly death or physical or sexual disability, against the background of some related cultural beliefs. The whole experience takes between a few minutes and one hour.

Whereas, in Koro-like syndromes, the underlying condition for the cultural myth is lacking; the development of the condition is insidious rather than acute, and fears regarding death are lacking, and may also be associated with other psychiatric conditions, for example, schizophrenia, affective disorder, and drug-taking for recreational or habitual reasons [5]. This report discusses three cases of male Koro syndrome, contributing to the existing literature on this phenomenon.

The cases described in this report likely represent modern or secondary forms of Koro rather than the classical epidemic type historically described in Southeast Asia. Contemporary literature views Koro as a culture-bound or culture-related syndrome that can manifest as an anxiety or somatoform disorder with genital preoccupation and fear of retraction or death. Such presentations are often influenced by sociocultural beliefs, personal stressors, and underlying anxiety vulnerability. Recognizing these variants as part of a broader Koro spectrum enhances understanding of their phenomenology and promotes culturally sensitive diagnosis and management.

## Case presentation

Case1

A 22-year-old unmarried male who worked in a deep freezer outlet in an Uttar Pradesh factory came to the psychiatry outpatient department (OPD) with ongoing anxiety and fear that his penis was eroding into his abdomen and becoming weaker over the previous two years. It was linked to severe anxiety, to the point where he couldn't sleep at night and kept thinking about it. The patient quit his job after a year because he blamed this finding on working in a deep freezer outlet in below-freezing temperatures. Even after leaving the job, the symptoms continued to interfere with his everyday functioning to the point where he used to have compulsive urges to pull out his genitalia to alleviate his anxiety.

There was no history of mental illness in the patient. The patient was clearly upset and nervous during the interview. As per the mental status evaluation, he was well-groomed, spoke softly, and described himself as having an anxious mood with a communicative and anxious affect. He kept thinking about his genitalia retracting into his stomach. The patient stated these thoughts to be his own and did not attempt to control them. The liver and kidney function tests, complete blood count, urine routine, microscopic examination, magnetic resonance imaging, and a pelvic ultrasound were performed to rule out infection and inflammatory processes and were found to be unremarkable. The thoughts were not intrusive or undesired, unlike obsessions, nor did they have a morbid origin, unlike delusions. The origin also stemmed from working in cold climates and cultural beliefs that were secondary to the negative health effects of low temperatures. Koro syndrome was thus diagnosed based on the patient's history, clinical examination, mental status examination, and the characteristic presentation of intense fear of genital retraction; this corresponds to a diagnosis of F48.8 (other specified neurotic disorders) in the International Classification of Diseases, Tenth Revision. The Diagnostic and Statistical Manual of Mental Disorders, Fifth Edition, Text Revision (DSM-5-TR), does not specifically diagnose Koro syndrome [6]; however, it can be categorized under other specified anxiety disorders with a specifier for culture-related anxiety disorder.

The Hamilton Anxiety Rating Scale (HAM-A) questionnaire revealed a score of 25, meaning moderate to severe anxiety [7]. The management focused on both acute management of his anxiety and the long-term management of Koro syndrome. In addition to cognitive behavioral therapy and supportive psychotherapy, the patient was started on tablet clonazepam 0.25 mg twice daily and tablet escitalopram 10 mg at night. The patient and his family received psychoeducation to help them comprehend the nature of the disorder. Additionally, relaxation methods like mindfulness and deep breathing exercises were explained. The patient was followed up in the OPD after a two-week and a one-month period, where he reported marked relief in symptoms.

Case 2

A 27-year-old, married IT professional presented to the psychiatry OPD with a 6-month history of anxiety and a persistent fear that his genitals were shrinking and being pulled into his abdomen. The symptoms were associated with intense anxiety attacks, particularly while cycling, necessitating stops by the roadside to relieve his distress. The symptoms significantly affected his occupational performance and interpersonal relationships, particularly with his spouse. He reported a stressful work environment and feelings of guilt related to frequent masturbation (approximately two to three times daily), which he believed had caused his condition. Consequently, he avoided any form of physical intimacy with his wife. There was no history of psychiatric illness, substance use, or significant medical comorbidity.

On mental status examination, the patient was well-groomed, cooperative, and spoke softly with coherent, relevant speech. His mood was anxious, with a communicative affect. He expressed persistent fears of genital retraction, acknowledging that these thoughts were his own but finding them difficult to dismiss. There were no psychotic features or delusional content. His insight was partial.

A complete blood count, liver and kidney function tests, urine analysis, and microscopic examination revealed no abnormalities. Ultrasonography of the pelvis and magnetic resonance imaging of the brain ruled out organic causes or intracranial pathology. On the HAM-A, the patient scored 27, indicating moderate to severe anxiety [7]. Based on his clinical presentation, absence of psychosis, and cultural explanations for his fears, a diagnosis of Koro syndrome (F48.8 - Other specified neurotic disorders, ICD-10) was made.

Treatment consisted of tablet paroxetine 12.5 mg once daily, tablet clonazepam 0.25 mg twice daily, and tablet buspirone 5 mg twice daily, along with psychoeducation, supportive psychotherapy, and cognitive-behavioral therapy focusing on anxiety management and reassurance. The patient was also taught relaxation and mindfulness techniques to manage anxiety-provoking thoughts. At two-week follow-up, the patient reported a marked reduction in anxiety and improved insight regarding the benign and psychogenic nature of his symptoms.

Case 3

The medicine department referred a 26-year-old single man who worked as a daily wage laborer in Uttar Pradesh to the psychiatry OPD after appropriate medical evaluation and management of dysuria and cloudy urine. He had been suffering from a distressing and persistent belief for a month that his penis was shrinking into his abdomen, accompanied by severe anxiety and a fear of death since the onset. He reported that he often checked his genitals and would ask his friends for help, too. His friends advised him to put cow dung on his genitals to keep them from retracting as an indigenous remedy. After a week of compliance with the remedy, he started having dysuria and cloudy urine, for which he went to the medicine OPD. The physical examination did not reveal any structural problems, but because of the strange complaints, he was sent for a psychiatric consult. During the outpatient psychiatric evaluation, the patient exhibited anxiety. There was no history of head injury, seizures, substance abuse, or the use of psychotropic medication. Collateral information from his relatives and examination of available case records did not disclose a previous history of psychiatric disorder. His socio-occupational functioning had been moderately impaired, as evidenced by reduced work attendance, social withdrawal, and difficulty performing daily activities due to his preoccupation with genital retraction.

His score on the HAM-A was 20, which meant he was moderately anxious [7]. He exhibited anxious affect, a preoccupation with the belief that his penis had diminished in size, and a lack of insight regarding the psychological origins of his symptoms during the mental status examination. There were no signs of psychosis, mood disorder, or obsessive-compulsive disorder. A diagnosis of Koro syndrome was made. The patient was started on tablet paroxetine 12.5 mg before bed and 0.25 mg clonazepam tablet as needed for acute anxiety. The focus of supportive psychotherapy and psychoeducational sessions was on cultural myths, sexual health, and how to deal with stress. A follow-up after one month indicated his anxiety was better, and he had a better understanding of his condition. He was also gradually able to return to his usual routine.

## Discussion

Koro syndrome is a culture-bound condition primarily identified in Southeast Asia, including India and China, characterized by an intense fear of genital retraction into the body, accompanied by anxiety and a belief in potentially fatal outcomes [8,9]. Historically linked to epidemic outbreaks, recent literature emphasizes isolated cases emerging in psychiatric clinics, especially within culturally sensitive environments [10]. The three cases in this report demonstrate the varied clinical manifestations and cultural precipitating factors of Koro syndrome in North India. All three patients were young adult males, aligning with previous epidemiological studies indicating that Koro mostly affects younger men [8,11]. In all instances, the clinical presentation was characterized by acute anxiety, panic-like symptoms, reassurance-seeking behavior, and deficits in social and occupational functioning. This finding is consistent with prior research that identifies panic-like anxiety, catastrophic misinterpretation of genital sensations, and body image-related fears as the principal psychopathological processes in Koro syndrome [2,9]. Each case demonstrates distinct cultural and psychosocial triggers. The initial patient ascribed his symptoms to exposure to cold environments, aligning with previous accounts that suggest somatic misinterpretations of environmental factors [11]. The second patient linked his symptoms to masturbation and sexual guilt, a recurrent theme observed in Asian populations where cultural myths surrounding semen loss and masturbation are prevalent [12]. The third patient tried a folk remedy (putting cow dung on his skin), which shows how community and peer pressure can make people stick to detrimental ways of thinking [9,13]. These differences show how the sociocultural context affects both the content and the way Koro presentations are made. Nosologically, Koro remains a subject of contention. In ICD-10, it is classified as F48.8 (other specified neurotic disorders) [14]. The DSM-5 does not offer a distinct diagnosis for Koro; instead, it categorizes it under culture-related syndromes, indicating its potential overlap with other anxiety or somatic symptom disorders [15]. ICD-11 adopts a more comprehensive approach by categorizing Koro as a manifestation of “cultural concepts of distress,” recognizing that these phenomena may not be limited to particular geographical areas but instead emerge from interactions among culture, mind, and body (6). This nosological uncertainty prompts inquiry into whether Koro constitutes a distinct entity or a culture-specific expression of underlying anxiety or body dysmorphic disorder [10,12]. The treatment for all three patients included selective serotonin reuptake inhibitors (SSRIs; escitalopram, paroxetine) along with benzodiazepines or buspirone for short-term anxiety relief, as well as psychotherapy and psychoeducation. As previous studies have shown, supportive psychotherapy, cognitive behavioral techniques, and correcting myths were all necessary to help patients deal with their cultural beliefs and stop them from making catastrophic misunderstandings [9,12,13]. During the short-term follow-up, all three patients exhibited partial to significant enhancement in anxiety and functional recovery. These results demonstrate the significance of amalgamating psychopharmacology with culturally attuned psychoeducation in the treatment of Koro syndrome. There are some limitations with this case series. The sample size in this report is limited and derived from a single tertiary care institution, which restricts the generalizability of findings. Additionally, long-term follow-up data were not systematically assessed. Despite these limitations, this case series contributes to the clinical understanding of Koro syndrome by illustrating its contemporary, culture-related presentations in North India. These findings emphasize that Koro may not only manifest as epidemic outbreaks but also as isolated, modern variants shaped by sociocultural factors, anxiety vulnerability, and individual psychosocial stressors.

## Conclusions

Koro syndrome, historically regarded as a culture-bound condition, continues to manifest sporadically within contemporary clinical practice. The three cases presented herein illustrate modern or secondary variants of Koro, characterized by genital retraction fears embedded within individual psychosocial stressors and sociocultural frameworks, rather than the epidemic form described in classical literature. Each case demonstrates how factors such as occupational strain, guilt related to masturbation, and traditional explanatory beliefs can shape the clinical presentation and trajectory of the disorder. All patients exhibited significant anxiety and functional impairment, reinforcing the importance of clinicians recognizing such culturally mediated syndromes in diverse psychiatric settings. Effective management required an integration of pharmacotherapy, psychoeducation, and culturally sensitive psychotherapy. Although constrained by a small sample size and limited follow-up, this case series contributes to the ongoing understanding of Koro and its contemporary forms. It emphasizes the relevance of incorporating cultural and psychological contexts into psychiatric assessment and treatment, thereby aligning with contemporary nosological perspectives on culture-related anxiety syndromes.
